# Comparative Proteomics Analysis Reveals Unique Early Signaling Response of *Saccharomyces cerevisiae* to Oxidants with Different Mechanism of Action

**DOI:** 10.3390/ijms22010167

**Published:** 2020-12-26

**Authors:** Prajita Pandey, Khadiza Zaman, Laszlo Prokai, Vladimir Shulaev

**Affiliations:** 1 Department of Biological Sciences, College of Arts and Sciences, University of North Texas, Denton, TX 76203, USA; PrajitaPandey@my.unt.edu; 2 Advanced Environmental Research Institute (AERI), University of North Texas, Denton, TX 76203, USA; 3 Department of Pharmacology and Neuroscience, University of North Texas Health Science Center, Fort Worth, TX 76107, USA; Khadiza.Zaman@unthsc.edu (K.Z.); Laszlo.Prokai@unthsc.edu (L.P.)

**Keywords:** oxidative stress, reactive oxygen species, antioxidants, global untargeted proteomics, TOR signaling, RAN signaling, MAPK cascade

## Abstract

The early signaling events involved in oxidant recognition and triggering of oxidant-specific defense mechanisms to counteract oxidative stress still remain largely elusive. Our discovery driven comparative proteomics analysis revealed unique early signaling response of the yeast *Saccharomyces cerevisiae* on the proteome level to oxidants with a different mechanism of action as early as 3 min after treatment with four oxidants, namely H_2_O_2_, cumene hydroperoxide (CHP), and menadione and diamide, when protein abundances were compared using label-free quantification relying on a high-resolution mass analyzer (Orbitrap). We identified significant regulation of 196 proteins in response to H_2_O_2_, 569 proteins in response to CHP, 369 proteins in response to menadione and 207 proteins in response to diamide. Only 17 proteins were common across all treatments, but several more proteins were shared between two or three oxidants. Pathway analyses revealed that each oxidant triggered a unique signaling mechanism associated with cell survival and repair. Signaling pathways mostly regulated by oxidants were Ran, TOR, Rho, and eIF2. Furthermore, each oxidant regulated these pathways in a unique way indicating specificity of response to oxidants having different modes of action. We hypothesize that interplay of these signaling pathways may be important in recognizing different oxidants to trigger different downstream MAPK signaling cascades and to induce specific responses.

## 1. Introduction

Oxidative stress indicates an imbalance between the production of reactive oxygen species (ROS) and a biological system’s ability to detoxify ROS and repair the subsequent damage [1]. Multiple ROS, including hydrogen peroxide (H_2_O_2_), hydroxyl radical (OH^•^), singlet oxygen, and superoxide anion (O2^•−^), are produced in biological systems and damage cellular components, including DNA, proteins, lipids, and carbohydrates [2] Cells constantly generate ROS in the mitochondria, endoplasmic reticulum (ER), plasma membrane and cytoplasm during aerobic respiration [3]. Under normal conditions, 1–2% of electrons leak from the mitochondrial electron transport chain and form a superoxide anion (O_2_^•−^) by cycling ubiquinol in the inner mitochondrial membrane [4]. ROS can overwhelm the cellular antioxidant defense system, either through an increase in ROS levels or a decrease in the cellular antioxidant capacity, leading to oxidative stress. Perturbations to the normal redox state of cells can result in toxic effects through ROS production leading to damage of cellular components [2]. Oxidative stress has been implicated in mediating programmed cell death (PCD) [5] and numerous diseases including cancer, cardiovascular disease, atherosclerosis, diabetes, arthritis, neurodegenerative disorders, and pulmonary, renal, and hepatic diseases [6]. Organisms have evolved a variety of specific mechanisms to protect themselves from the damaging effect of different ROS. The damage of a cell’s macromolecular structures activates antioxidant defense pathways aimed to regulate cellular redox balance [7]. While in some cases a particular ROS species was linked to a specific pathology, the connection between individual ROS species and different diseases remains poorly understood. To complicate the story even more, a significant body of evidence suggests that in addition to cytotoxicity, ROS compounds have an important signaling role [6,7].

Despite the long history of oxidative stress research, very little is known about early signaling events involved in oxidant recognition that trigger a specific response. Early ROS signaling can manifest itself in changes in transcription, protein expression, post-translational modifications, and perturbation of metabolic networks. Global approaches, such as genomics, transcriptomics, proteomics and metabolomics, have enabled investigation of complex molecular networks involved in oxidative stress response [7]. Several studies have focused on quantifying global changes in transcriptome, proteome and metabolome following treatment with various oxidants.

Despite these studies shedding light on some regulatory responses, early signaling events in stress response that trigger specific defensive mechanisms to a given oxidant, which can make the difference between survival and death, are still largely unknown. This calls for a comprehensive study of the molecular mechanisms involved in survival, repair of damage, or cellular recovery from damage related to oxidative stress induced by oxidants with a diverse mechanism of action.

*Saccharomyces cerevisiae* is a useful eukaryotic model for studying various aspects of oxidative stress at biochemical, cellular, and molecular levels. The nature of stress factors in yeast, and the damage caused by the oxidative stress to nucleic acids [8], lipids [9], proteins [10], and other cell components are like higher eukaryotes. The primary defenses involved in the catabolism of ROS and the scavenging system is also similar to higher eukaryotes [11]. They acquire multiple H_2_O_2_ detoxifying enzymes, such as catalases, cytochrome *c* peroxidase, glutathione peroxidases, glutaredoxins and peroxiredoxins, many in distinct cellular compartments, which makes it an advanced model for the analysis of oxidative stress [12]. Additionally, a completely sequenced genome, easy laboratory handling and the availability of a nearly complete collection of gene deletion mutants is a big advantage to the research [13]. They can be manipulated physiologically in different growth and environmental conditions [13].

The objective of our study is to determine early signaling events required for sensing different oxidants and triggering oxidant-specific defenses. Additionally, we aimed to identify distinct mechanisms corresponding to a particular oxidant. In efforts to delve into the distinct molecular mechanisms in oxidative stress response, we designed a comparative LC-MS/MS-based proteomic workflow relying on a hybrid mass spectrometer equipped with a high-resolution mass analyzer (Orbitrap) to find changes in the *S. cerevisiae* proteome after exposure to chemically diverse ROS generating agents with distinct mechanism of action: H_2_O_2_, menadione (superoxide-generating agent), cumene hydroperoxide (CHP, an aromatic hydroperoxide), and diamide (thiol oxidant).

## 2. Results and Discussion

### 2.1. Changes in Global Proteome Expression Caused by Different Oxidants

Oxidative stress can be induced by different oxidants with different molecular targets. It has been shown that cells have distinct mechanisms to maintain protection against different ROS utilizing different sets of oxidative-stress-response genes [14]. Several studies also show that different oxidants induce differential transcriptional, translational, and metabolic response [14,15], but the precise mechanism of sensing different oxidants and triggering oxidant-specific defenses remains unknown.

Our label-free global quantitative proteomics analysis based on high-resolution mass spectrometry (Orbitrap) revealed that the yeast proteome responds to oxidants with different mechanisms of action. We identified important signaling cascades that are triggered as early as 3 min in response to stress. These early stress-responsive mechanisms may be very important to determine the fate of the cells after stress. To identify early changes in proteome induced by different oxidants, we used four diverse ROS generating agents with distinct mechanisms of action, including H_2_O_2_, menadione (superoxide inducer), cumene hydroperoxide (CHP, an aromatic hydroperoxide), and diamide (thiol oxidant).

The total number of proteins identified in each individual treatment is shown in Supplementary Table S2. Quantitative analysis identified 196 proteins that significantly changed their expression in response to H_2_O_2_, 569 proteins in response to CHP, 369 proteins in response to menadione and 207 proteins in response to diamide (Supplementary Table S3).

**Figure 1:**
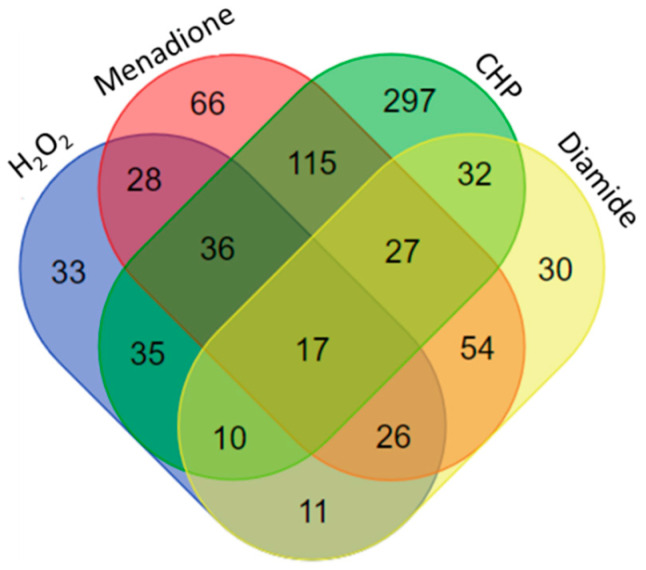
Venn diagram showing unique and shared proteins significantly regulated (*p* < 0.05) by H_2_O_2_, hydroperoxide (CHP), menadione, and diamide 3 min post treatment. Data was analyzed using Proteome Discoverer 2.4 using label-free quantification relying on precursor ions of tryptic peptides based on high-resolution (Orbitrap) mass spectrometry.

Moreover, comparison of response to different oxidants showed that each oxidant triggered a unique response on a protein level as early as 3 min post treatment with a set of proteins that respond only to each individual oxidant. Comparisons of unique and shared proteins between treatments are shown in Figure 1. Only 17 proteins were common across all four treatments, but many proteins were shared between two or three oxidants. CHP-treatment had largest number of unique regulated proteins (297) followed by menadione (66), H_2_O_2_ (33) and diamide (30) and A complete list of proteins is given in Supplementary Table S3.

### 2.2. Major Regulatory Pathways Involved in Oxidative Stress Response

The molecular and cellular functions most significantly impacted by different oxidants are summarized in Table 1. Several perturbed molecular and cellular functions were found to be shared between all four or fewer treatments, suggesting a varied extent of overlap in the oxidative stress response. This correlates with previous findings that there is overlap in stress response after treating *S. cerevisiae* with various oxidants [14]. Although some processes were similar amongst the four treatments, the overall associated molecular and cellular functions appeared to be different (Table 1). Our data indicate that the global early response to oxidative insult involves the activation of repair and survival mechanisms, which is not surprising (Table 1). The most significant molecular and cellular function in response to H_2_O_2_, diamide and menadione was cell death and survival, whereas gene expression was the most significant in response to CHP. Although yeast has a pool of antioxidants and pro-survival pathways protecting against ROS, these systems may fail in the case of sudden or prolonged insult by ROS [16], which impacts cell death and survival pathways. Drug metabolism and small molecule biochemistry were unique to H_2_O_2,_ while DNA replication, recombination, and repair were unique to CHP, and protein synthesis was unique to menadione. RNA post-transcriptional modification was regulated by all treatments, suggesting its importance in oxidative stress response. Recent studies have revealed post-transcriptional modifications as important biomarkers of ageing and age-related diseases [17].

**Figure 2:**
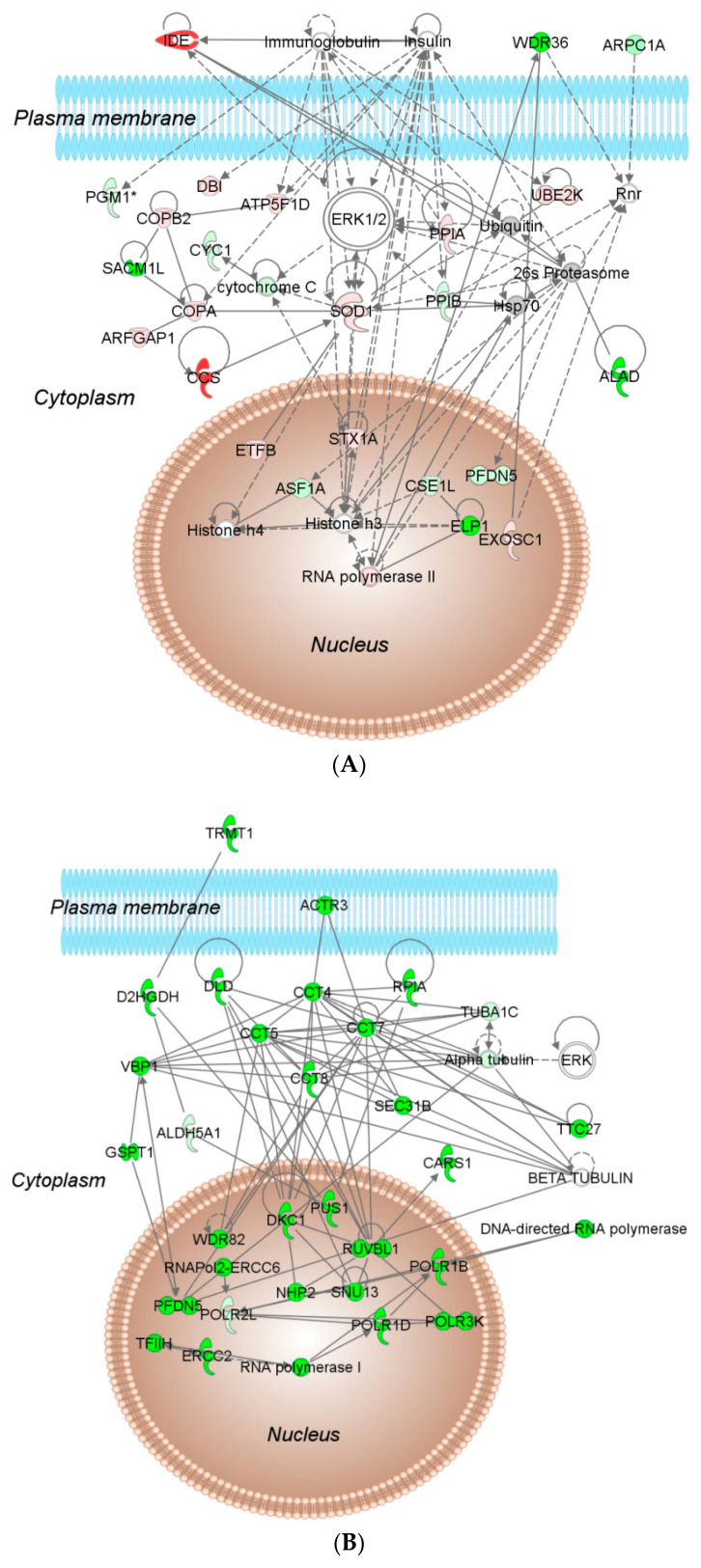

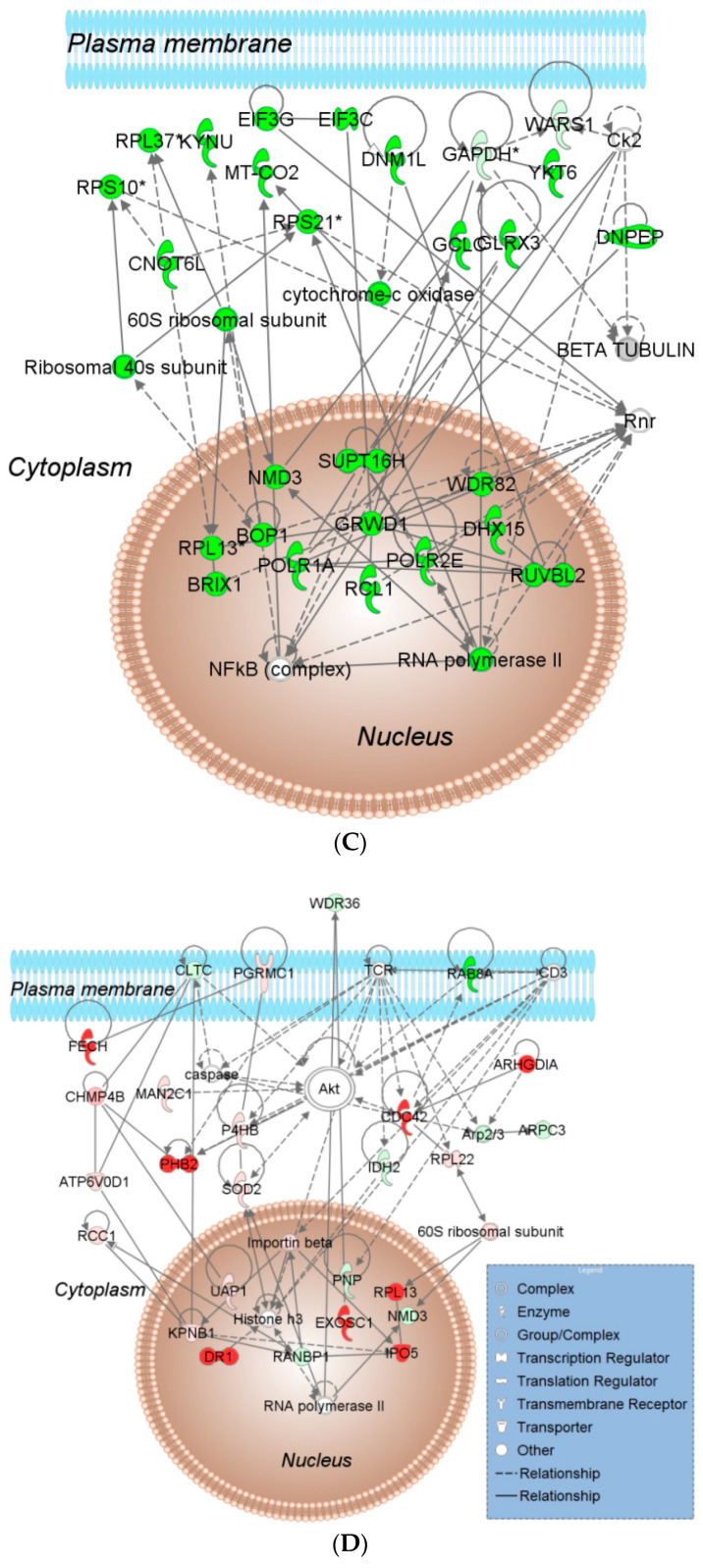
Top networks in *S. cerevisiae* response to oxidants at 3 min by (**A**) H_2_O_2_: cell death and survival, drug metabolism, small molecule biochemistry; (**B**) CHP: cellular assembly and organization, cell-to-cell signaling and interaction, reproductive system development and function. Top networks in *S. cerevisiae* response to oxidants at 3 min by (**C**) menadione: protein synthesis, cancer, cell death and survival; and (**D**) diamide: cell cycle, infectious diseases, cellular assembly and organization. The shapes (see legend in blue box) represent molecular classes of the regulated proteins. In the network, red and green colors denote upregulation and downregulation in response to the four treatments, respectively. The intensity of color indicates the relative magnitude of fold change in the protein expression pattern. Solid and dashed lines represent direct and indirect interactions, respectively.

Furthermore, IPA^®^-based network analysis identified associated network functions regulated by different oxidants (Figure 2, Supplementary Table S4). The highest scoring associated network function with the largest number of molecules in response to H_2_O_2_ was cell death and survival, drug metabolism, small molecule biochemistry (Figure 2A). In response to CHP, the highest scoring associated network functions were cellular assembly and organization, cell-to-cell signaling and interaction, reproductive system development and function (Figure 2B). In response to menadione, the highest scoring associated network functions were protein synthesis, cancer, cell death and survival (Figure 2C). In response to diamide, the highest scoring associated network functions were cell cycle, infectious diseases, cellular assembly and organization (Figure 2D). Both menadione and H_2_O_2_ had cell death and survival as top perturbed network functions. Not surprisingly, many of these pathways have been associated with oxidative stress [18,19,20]. Another important observation was downregulation of cytochrome *c* oxidase in cell death and survival pathway in response to H_2_O_2_ and menadione. The cell death in yeast was linked to the release of cytochrome *c* from the outer mitochondrial membrane to the cytoplasm, leading to disruption of oxidative phosphorylation [21].

Using IPA^®^, we also interrogated canonical pathways significantly responding to oxidant treatment (Table 2). Similar to network functions, the four oxidants induced different sets of pathways, though some of the pathways were induced by multiple oxidants. Specifically, TOR (target of rapamycin) signaling, nicotinamide adenine dinucleotide (NAD) biosynthesis II (from tryptophan), glycogen degradation and regulation of eIF4 (eukaryotic initiation factor 4) signaling were unique in response to H_2_O_2_, glycine cleavage complex was unique in response to CHP, RhoA signaling and heme biosynthesis II were unique in response to menadione, and sumoylation pathway, and urate biosynthesis/inosine 5′-phosphate degradation were unique to diamide-treated cells.

**Figure 3:**
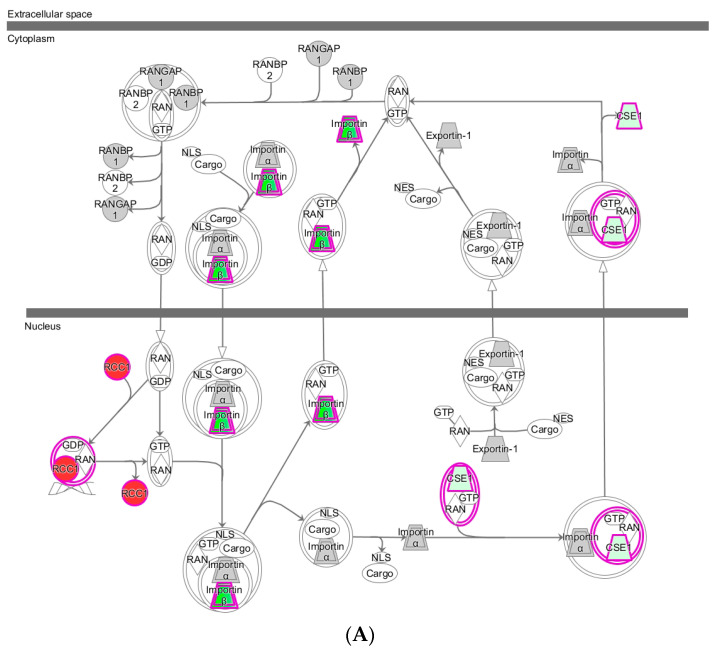

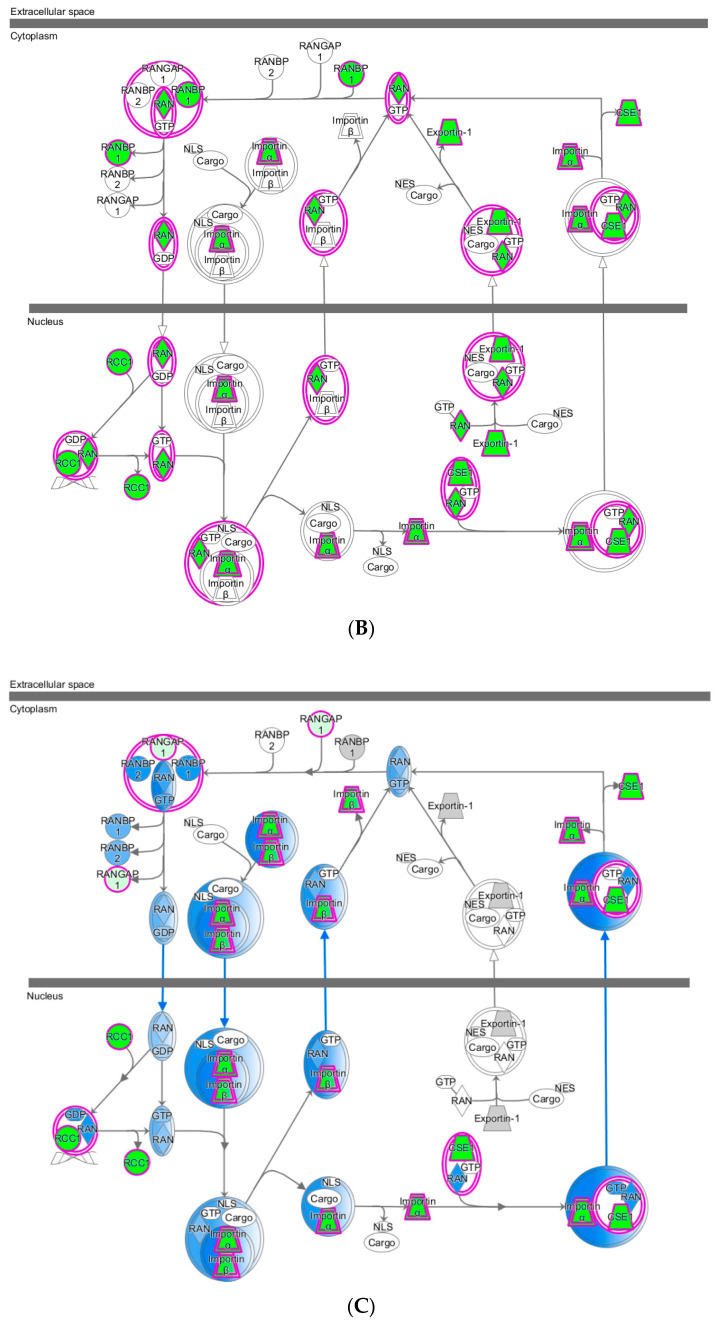

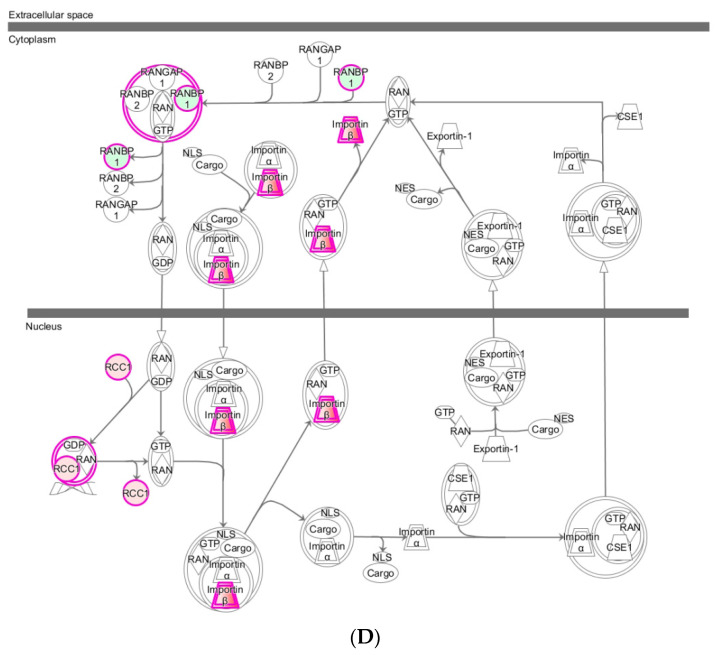
Regulation of the canonical Ran signaling pathway in *S. cerevisiae* response to oxidants at 3 min by (**A**) H_2_O_2_ and (**B**) CHP. Regulation of the canonical Ran signaling pathway in *S. cerevisiae* response to oxidants at 3 min by (**C**) menadione and (**D**) diamide. Networks were constructed using IPA^®^ based on global untargeted proteomics analysis using label-free quantification relying on precursor ions of tryptic peptides based on high-resolution (Orbitrap) mass spectrometry. Regulation of the pathways and individual components are indicated by color: green for protein downregulation, red for protein upregulation, blue for pathway inhibition, and orange for pathway activation.

Among the canonical pathways regulated by oxidants several signaling pathways, including Ran (Ras-related nuclear protein), TOR, Rho, and eIF2 (eukaryotic initiation factor 2) signaling, were previously implicated in stress response, but their precise role in different oxidant recognition and downstream signal transduction remained elusive [22,23,24]. We observed however that Ran signaling pathway significantly enriched in all treatments to a different degree, suggesting a different mode of action (Figure 3). Ran GTPase is a guanosine triphosphate (GTP) binding protein involved in nucleocytoplasmic transport [25]. The *S. cerevisiae* homologue of the Ran GTPase is Gsp1p [25]. Two nucleotide forms of Ran (RanGTP and RanGDP) generate a gradient between the nucleus and cytoplasm [26]. The nucleocytoplasmic transport controls signal transduction, gene expression, cell-cycle progression, apoptosis, and regulation of stress response [27]. It also regulates ubiquitin-mediated protein degradation during the cell cycle [28]. Ran GTPase requires localization to the nucleus and GTP loading by the chromatin-associated exchange factor RCC1, a protein that promotes the exchange of Ran-bound guanosine diphosphate (GDP) by GTP [29]. Despite Ran signaling not been extensively studied in response to different oxidants, several studies outlined its role in response to H_2_O_2_ [22]_._ H_2_O_2_-induced oxidative stress has shown to collapse the nucleocytoplasmic Ran GTPase gradient in growing cells, leading to inhibition of the nuclear import [30]. Sensitivity to oxidative stress causes redistribution of the small GTPase Gsp1p/Ran from the nucleus to the cytoplasm, causing nuclear import inhibition [31]. This leads to elevation of cytoplasmic GTPase levels. Small GTPase, Gsp1p/Ran, also plays a major role in nuclear localization of Yap1p (AP-1-like transcription factor), which is a transcription factor essential for oxidative stress response in yeast [32]. Our results also show upregulation of the SRM1 (nucleotide exchange factor for Gsp1p) by diamide and H_2_O_2_ and downregulation by menadione and CHP.

It has been previously shown that oxidative stress causes oxidation of cysteine residues in RCC1, which can disrupt the Ran signaling [22]. Cysteine oxidation is a well-established biomarker of oxidative stress. Oxidation of cysteine residues can change the function of a protein in networks of kinases, phosphatases and apoptotic cascades, and can cause changes in transcriptional activity [33]. Hence, additional studies on posttranslational modifications, including oxidative posttranslational modifications, are required to further understand the role of Ran signaling in early response to different oxidants.

TOR signaling in *S. cerevisiae* is involved in cell growth, ribosome biogenesis, translation initiation, metabolism, stress response, aging, and autophagy [34]. TOR is a Ser/Thr-protein kinase that is evolutionary conserved [35]. The cells lacking TORC1 have shown sensitivity to oxidative stress, suggesting TOR signaling plays an important role in stress response [23]. The other important role of TOR signaling is the transcriptional regulation of MSN2/4, an important transcription factor in response to oxidative stress [23]. We observed significant regulation of TOR signaling in all four oxidant treatments (Supplementary Figure S1).

We found that a significant response of TOR signaling was associated with downregulation of several protein components of the small (40S) ribosomal subunits. The largest number of downregulated components of the small (40S) ribosomal subunits was observed in CHP treatment. Our result is consistent with the established role of TOR signaling in ribosome biogenesis [34]. Regulation of TOR signaling by ROS could contribute to alteration of cell viability, cell cycle progression and translation initiation.

### 2.3. MAPK Cascade Module and Signal Transduction

Cells acquire various signal transduction pathways to transmit external molecular signals effectively into the cell [36]. Eukaryotic cells have a conserved module composed of three protein kinases; the mitogen-activated protein kinase (MAPK) cascade to transduce the extremal stimuli [37]. The three kinases are: MAPK (also known as extracellular signal-regulated kinase (ERK)), MAPK kinase (MAPKK, also known as mitogen-activated, ERK-activating kinase (MEK)), and MAPK kinase kinase (MAPKKK, also known as MEK kinase (MEKK)) [37]. Many components of the MAPK module and their functions found in *S. cerevisiae* are conserved in eukaryotes [38]. MAPK cascades regulate many physiological processes such as the life cycle of budding and fission yeasts, pheromone response pathway for conjugation and meiosis, high-osmolarity sensing pathway for osmotic stress, hypo-osmolarity and heat-sensing pathway for cell wall biosynthesis [36]. MAPK cascades also activate a subset of the components of the pheromone response pathway for pseudohyphal growth [36].

During oxidative stress, Rho1 GTPase regulates cell wall integrity (CWI) by activating the MAPK cascade for CWI signaling. This is the main route responsible for maintaining homeostasis in the cell wall [39]. Guanosine dissociation inhibitor (GDI) proteins play a major role in controlling both the timing and the localization of Rho1 GTPase activity. Cell surface sensors such as Wsc1, Mlt1 and Mid2 interact with Rom2 and activate Rho1 as a stress response [24]. This activates yeast protein kinase C (Pkc1) and Pkc1 phosphorylates MAPKKK Bck1 trigger the MAPK module. This phosphorylation stimulates Mkk1 and Mkk2, which phosphorylate and activate Slt2/Mpk1. Cyclin dependent protein kinase (CDK) at the G1–S transition [40] and polo kinase are also responsible in activating Rho1 [41]. Rho1 activity is dependent on TORC1 signaling [42] TORC2 signaling [43], and phospholipid metabolism [44]. It has also been found that oxidative stress may prevent activation of pheromone signaling and high-osmolarity glycerol response (HOG) pathways, suggesting crosstalk mechanisms between them [45].

**Figure 4:**
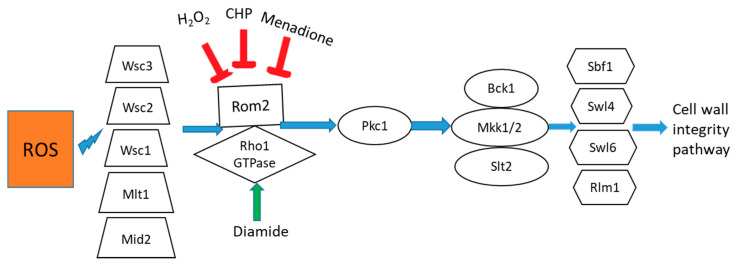
Model for how different oxidants affect the Rho protein signal transduction in *S. cerevisiae* at 3 min. Menadione, CHP and H_2_O_2_-induced oxidative stress disrupt the signal transduction, causing defective cell wall homeostasis. Symbols: protein kinases, ovals; guanosine triphosphate (GTP)-binding proteins, diamonds; scaffold, adaptor, and activating proteins, rectangles; cell surface proteins, trapezoids; transcription factors, hexagon; activation, arrows; inhibition, T-bars.

Interestingly, we observed Rho signaling significantly altered in all the treatments. However, their regulation differed amongst the treatments. In H_2_O_2-_treated cells, there was a downregulation of Rho GDP-dissociation inhibitor, suggesting disruption of the GDP/GTP exchange reaction of the Rho proteins by facilitating the dissociation of GDP from them, and the obstructing of subsequent binding of GTP. Furthermore, depletion of the Rho GDP-dissociation inhibitor results in degradation of Rho1 and cell division cycle protein 42 (Cdc42) by the proteasome [46]. In menadione-treated cells, there was downregulation of GTP-binding protein Rho3. This phenomenon is also linked to slow growth (Figure S2), as Rho3 are known to regulate polarized secretion and the actin cytoskeleton [47]. In CHP-treated cells, we observed downregulation of the GTP-binding protein Rho3, GTP-binding protein Rho1 and Rho GDP-dissociation inhibitor, also suggesting disruption of Rho protein signal transduction. In diamide-treated cells, there was upregulation of Rho1 GDP-GTP exchange protein 2 and Rho GDP-dissociation inhibitor, suggesting induced Rho signaling in response to diamide. Our result indicated that H_2_O_2_, menadione and CHP compromised the overall cell wall integrity; however, diamide stimulated downstream signaling pathway (Figure 4). As mentioned above, there are crosstalks between these pathways. Oxidative stress was shown to disrupt recruitment of (Ste5) and its associated proteins to the plasma membrane and reduce nuclear translocation of the MAPK protein Hog1, causing inhibition of pheromone signaling pathways and the HOG pathway, respectively [45]. We observed that Hog1 was downregulated in all four treatments, which infers the interference in the HOG pathway.

Although there is a vast body of research on oxidative stress in biological systems and the role it plays in various diseases, several aspects of the oxidative stress response remain elusive [1,2,3,4,7,8,9,10,11,12,13,14,48,49,50,51,52]. Very little is known about early signaling events in stress response that trigger specific defensive mechanisms to a given oxidant, which can make the difference between survival and death. It has been proposed that early transcriptional events, changes in protein expression and patterns of posttranslational modifications, along with rapid changes in the metabolome, coordinate complex molecular networks controlling rapid reprogramming of cellular metabolism and activation of defense pathways [53,54,55,56,57,58]. A detailed understanding of these processes is required to design new approaches for combating stress-induced pathologies.

Current understanding of oxidative stress responses in yeast has mostly derived from studies focused on later time points. It was shown that gene expression in stress response as early as 30 s [46]; therefore, it is essential to investigate early responses to oxidative stress. The early signaling mechanism(s) that control oxidant perception and signal transduction leading to activation of the antioxidant defense response and survival mechanisms tailored toward specific oxidative insult is crucial for comprehensive understanding of oxidative stress and biochemical and signaling networks mediating stress response. Our results using label-free proteomics relying on precursor ions tryptic peptides quantified by high-resolution (Orbitrap) mass spectrometry have shown that specific proteome-level response is triggered, indeed, to different oxidants within minutes after treatment and several signaling networks are activated and may be responsible for furnishing oxidant specific defenses.

## 3. Materials and Methods

### 3.1. Yeast Strains and Culture Media

Yeast strain BY4743 ([4741/4742] MATa/MATα his3delta1/his3delta1 leu2delta0/leu2delta0 lys2delta0/+met15delta0/+ura3delta0/ura3-delta0) was obtained from American Type Culture Collection (ATCC, Manassas, VA, USA, Catalog#201390). Cultures were kept in long-term storage frozen at −80 °C in glycerol stocks. Work cultures were kept at 4 °C in Yeast Extract–Peptone–Dextrose (YPD) Medium (yeast extract 0.1% (*w*/*v*), peptone 0.5% (*w*/*v*) and dextrose 2% (*w*/*v*)) agar plates.

### 3.2. Cell Growth and Oxidative Stress Conditions

*S. cerevisiae* cells were initially batch grown overnight at 30 °C, pH 6.0, 150 rpm, in minimal medium (25 mL) containing Difco yeast nitrogen base without amino acids, 2% (*w*/*v*) dextrose supplemented with uracil 20 mg/L, L-leucine 60 mg/L and L-histidine 20 mg/l until mid-exponential phase was reached, based on measuring optical density measurement at 600 nm (OD_600_~1.5). The inoculum from overnight culture was used to inoculate minimal media (25 mL) containing Difco yeast nitrogen base without amino acids, 2% (*w*/*v*) dextrose supplemented with uracil 20 mg/L, L-leucine 60 mg/L and L-histidine 20 mg/L. The cultures were grown at 30 °C, pH 6.0, 150 rpm. Conditions of oxidative stress were achieved by the addition of 1.2 mM H_2_O_2_, CHP 190 µM, menadione 150 µM, and diamide 1.5 mM at the mid-exponential phase (OD_600_~1.5). Controls without CHP were made by adding the same volume of 95% (*v*/*v*) ethanol (the solvent for CHP). Controls without menadione were made by adding the same volume of 100% (*v*/*v*) DMSO (the solvent for menadione). Controls without H_2_O_2_ and diamide were not treated with any chemical, as their solvent is water. Three biological replicates were carried out for each condition.

### 3.3. Sample Collection and Processing

Samples were collected at 3 min after the addition of oxidants. To quench metabolism, cells were harvested in aqueous 40% ethanol (*v*/*v*) solution containing 0.8% NaCl (*w*/*v*), kept at −40 °C using a dry ice-ethanol bath, as described in Spura et al. [59]. Samples were centrifuged for 5 min at 1000× *g* and −10 °C. Temperature was monitored after centrifugation to ensure that it was below −35 °C. The supernatants were discarded and the pellets were suspended in 100% methanol, freeze-dried for 48 h using a Labconco 79480 Freeze Dry System (Labconco, Kansas City, MO, USA) and stored at −80 °C until use.

### 3.4. Protein Extraction and Digestion

Proteins were extracted using a lysis buffer made of 8 M urea in 25 mM ammonium bicarbonate One mL of lysis buffer was added to 35 mg (dry weight) of yeast cells and vortexed for a 1 min to ensure proper resuspension. Two hundred μL of 0.5 mm glass beads were added. The pellet was milled for 2 min at 30 Hz in the Retsch Mixer Mill MM 300 (Retsch GmbH, Haan, Germany) and kept in ice for 1 min. This step was repeated 5 times. Samples were centrifuged for 15 min at 20,000× *g* at 4 °C in a refrigerated microcentrifuge and the supernatant was transferred into a clean tube. Protein concentration was determined using Nanodrop 2000 (Thermo Fisher Scientific, Waltham, MA, USA) after calculating the appropriate volume containing 100 µg of protein, 25mM of ABC was added to bring the volume to 100 µL. The extracted proteins were reduced with dithiothreitol and incubated at 56 °C for 30 min and carbamidomethylated by addition of 200 mM iodoacetamide [16,60]. The samples were then diluted 9-fold with 25 mM ammonium bicarbonate and digested overnight at 37 °C with trypsin. The enzymatic digestion was then stopped by acidifying the sample with 15 μL of acetic acid.

### 3.5. Solid Phase Extraction (SPE)

The digested protein was cleaned up using a SOLA HRP cartridge (Thermo Scientific, Runcorn, Cheshire, UK). After activating and equilibrating the column bed with 500 µL of acetonitrile, the bed was equilibrated with 2× 500 µL of 0.1% TFA in water. The protein was eluted in 10 fractions using the elution solvents (2 × 250 µL) in the Supplementary Table S1. Fractions were dried in a vacuum concentrator and re-suspended in 150 μL of 5% acetonitrile and 0.1% formic acid (*v*/*v*) in water for LC-MS/MS analysis.

### 3.6. Data-Dependent NanoLC-MS/MS Analyses

The digested peptides were analyzed employing a hybrid linear ion trap (LTQ) Orbitrap Velos Pro mass spectrometer (Thermo Scientific, San Jose, CA, USA) equipped with a Proxeon (Odense, Denmark) nanoelectrospray ionization (NSI) source and connected to a nanoACQUITY UPLC System (Waters Corporation, Milford, MA, USA). A nanoEaseTM M/Z HSS T3 analytical column, 75 mm × 150 cm (Waters Corporation, Milford, MA; PN: 186008816) was used for peptide separation at 400 nL/min flow rate using 3-h linear gradient (from 5% to 95% B). The eluent system consisted of (A) 0.1% formic acid in water (*v*/*v*) and (B) 0.1% formic acid in acetonitrile (*v*/*v*). Peptides were eluted through a Picotip emitter (internal diameter 10 ± 1 µm; New Objective, Woburn, MA, USA) and directly sampled by the nano-electrospray source of the mass spectrometer in a positive ion mode. NSI spray voltage and capillary temperature maintained during the gradient run were 1.8 kV and 250 °C respectively.

FT full-scan mass spectra were attained at 60,000 mass resolving power (*m/z* 400) in the *m/z* range of 350 to 1650. Collision induced dissociation (CID) was achieved using helium in the linear ion trap to obtain MS/MS product ion scans of the top ten most abundant precursor ions from the full scan mass spectra using a 2.5 Da isolation width and normalized collision energy of 35%. The precursor ions selected for CID were then dynamically excluded from further MS/MS analysis for 30 s.

### 3.7. Data Processing and Statistical Analysis

MS and MS/MS data were analyzed using the SEQUEST HT algorithm in the Proteome Discoverer (version 2.4, Thermo Fisher Scientific, San Jose, CA, USA. Protein identification and quantification were conducted against the FASTA database composed of *S. cerevisiae* Uniprot protein database (6049 entries). SEQUEST search parameters included: peptide length > 5 amino acids, maximum of one tryptic missed cleavage events, cysteine carbamidomethylation as a fixed modification and oxidation of methionine and deamidation of glutamine and asparagine as variable modifications, precursor mass tolerance of 25 ppm and fragment mass tolerance of 0.80 Da. Peptide false discovery rate (FDR) was directly determined by the target-decoy strategy. PSMs and protein level FDR were both set at 1%. Total ion current (TIC) normalization signal to noise ratio was set to 2. Fold changes of the proteins were calculated from normalized precursor intensity values by comparing the treatment conditions to control. Student’s *t*-tests were used to determine statistically significant differential protein expressions (*p* < 0.05), and the fold change threshold was set to ±2.0. We uploaded our data to the ProteomeXchange Consortium [61] by the PRIDE partner repository (assigned dataset identifier: PXD022685).

### 3.8. Bioinformatics

Differentially expressed proteins from each oxidant group were submitted with UniProt identifiers and fold changes to Ingenuity Pathway Analysis^®^ (IPA^®^, QIAGEN, Redwood City, CA, USA) for core analysis. The matched proteins were used to generate molecular networks including canonical pathways and upstream regulatory analysis. The core analysis allowed us to delineate specific response to different oxidants. IPA^®^ also predicted possible upstream regulators of the proteins in this study, which were assigned as inhibited or activated according to the Z-score, a statistical result of differential protein expression according to the fold changes. IPA^®^ employs right-tailed Fisher’s exact test to calculate the overlaps of *p*-values [62].

## 4. Conclusions

In summary, our comparative proteomics analysis has revealed unique early signaling response of *Saccharomyces cerevisiae* to oxidants with different mechanism of action as early as 3 min after treatment. Our goal was to identify early signaling mechanisms involved in oxidant recognition and molecular switches that trigger differential response to oxidants with different mechanism of action.

We performed global untargeted proteomics to map out the proteome under oxidative stress generated by four distinct oxidative stress-inducing chemicals, namely H_2_O_2_, menadione, CHP, and diamide. We found that different oxidants elicit diverse proteomic responses, suggesting activation of different signal transduction pathways by each oxidant. According to our pathway analyses, these proteins participated in 25 different networks, and signaling pathways mostly regulated by oxidants at early time points were Ran, TOR, Rho, and eIF2. Furthermore, each oxidant regulated these pathways in a unique way indicating specificity in the response to different oxidants having different modes of action. Interplay of signaling pathway may be important in recognizing different oxidants and may trigger different downstream MAPK signaling cascades and induce specific response to each oxidant. Therefore, it is imperative to use a combination of genetic analysis and omics studies to identify the specific role of each pathway and individual signaling components regulated by different oxidants. Many identified proteins are subject to posttranslational regulation by phosphorylation and oxidative modification; therefore, more studies on the role of specific posttranslational modifications in early oxidative stress response signaling are required to further understand the specific signaling mechanism triggered by each oxidant. It is also important to study the differential dynamics of protein networks perturbed by each oxidant to identify downstream signaling events and to determine if any of these early responses are transient or have long-term impact on cellular functions.

## Figures and Tables

**Table 1:** Top five molecular and cellular functions represented by Ingenuity Pathway Analysis^®^ (IPA^®^) using significantly regulated proteins identified by label-free quantitative proteomics performed in *S. cerevisiae* strains treated with the chosen four different oxidants. Asterisks (*) indicate unique molecular and cellular function.

Treatment	Represented Process	*p*-Value Range	Number of Molecules
H_2_O_2_	Cell Death and Survival	4.39 × 10^−2^–3.63 × 10^−6^	22
	Drug Metabolism *	4.53 × 10^−2^–9.37 × 10^−6^	4
	Small Molecule Biochemistry *	4.53 × 10^−2^–9.37 × 10^−6^	23
	RNA Post-Transcriptional Modification	1.84 × 10^−2^–2.19 × 10^−5^	7
	Molecular Transport	4.53 × 10^−2^–5.60 × 10^−6^	10
CHP	Gene Expression	1.88 × 10^−3^–2.09 × 10^−13^	38
	RNA Damage and Repair	2.64 × 10^−8^–2.09 × 10^−13^	28
	RNA Post-Transcriptional Modification	4.70 × 10^−4^–2.09 × 10^−13^	39
	DNA Replication, Recombination, and Repair *	2.60 × 10^−3^–1.15 × 10^−12^	47
	Nucleic-Acid Metabolism	3.72 × 10^−3^–1.15 × 10^−12^	34
Menadione	Cell Death and Survival	2.78 × 10^−2^–1.67 × 10^−12^	46
	Gene Expression	2.39 × 10^−2^–2.46 × 10^−7^	39
	RNA Damage and Repair	2.21 × 10^−2^–2.46 × 10^−7^	17
	RNA Post-Transcriptional Modification	1.91 × 10^−2^–2.46 × 10^−7^	18
	Protein Synthesis *	1.33 × 10^−2^–1.69 × 10^−6^	39
Diamide	Cell Death and Survival	2.17 × 10^−2^–1.50 × 10^−9^	28
	Gene Expression	2.17 × 10^−2^–1.04 × 10^−6^	10
	RNA Damage and Repair	6.86 × 10^−3^–1.04 × 10^−6^	15
	RNA Post-Transcriptional Modification	9.35 × 10^−4^–1.04 × 10^−6^	14
	Molecular Transport	2.17 × 10^−2^–2.50 × 10^−5^	20

**Table 2:** Top five significant canonical pathways generated by IPA^®^ using significantly regulated proteins identified by label-free quantitative proteomics performed in *S. cerevisiae* treated with the four different chosen oxidants. Asterisks (*) indicate unique molecular and cellular function.

Treatments	Top Canonical Pathway	*p*-Value
H_2_O_2_	Ran Signaling	1.85 × 10^−5^
	mTOR Signaling *	4.79 × 10^−4^
	NAD Biosynthesis II (from Tryptophan) *	6.06 × 10^−4^
	Glycogen Degradation I *	6.06 × 10^−4^
	Glycogen Degradation II *	8.32 × 10^−4^
CHP	Protein Ubiquitination Pathway	1.27 × 10^−12^
	Ran Signaling	1.36 × 10^−8^
	eIF2 Signaling	1.50 × 10^−8^
	Remodeling of Epithelial Adherens Junctions	6.96 × 10^−6^
	Glycine Cleavage Complex *	2.19 × 10^−5^
Menadione	Ran Signaling	5.06 × 10^−10^
	Remodeling of Epithelial Adherens Junctions	3.48 × 10^−6^
	RhoA Signaling *	9.89 × 10^−6^
	Heme Biosynthesis II *	1.76 × 10^−5^
	Fcγ Receptormediated Phagocytosis in Macrophages and Monocytes *	2.28 × 10^−5^
Diamide	Ran Signaling	3.81 × 10^−7^
	Protein Ubiquitination Pathway	6.40 × 10^−5^
	Sumoylation Pathway&	5.57 × 10^−4^
	Urate Biosynthesis/Inosine 5′-phosphate Degradation *	1.00 × 10^−3^
	eIF2 Signaling	1.39 × 10^−3^

## Data Availability

Raw data were made available through the ProteomeXchange Consortium [61] by the PRIDE partner repository (assigned dataset identifier: PXD022685).

## Supplementary Materials

Supplementary materials can be found at https://www.mdpi.com/1422-0067/22/1/167/s1. Table S1: Elution solvents for high-pH reversed-phase fractionation of protein digests, Table S2: Lists of identified and validated proteins using Proteome Discoverer 2.4, along with their gene ontology (GO) annotations, Table S3: Lists of regulated *S. cerevisiae* proteins, Table S4: Top five protein networks generated by IPA^®^ using significantly regulated proteins identified by label-free quantitative proteomics performed in *S. cerevisiae* strains treated with the four different oxidants, Figure S1: TOR signaling in *S. cerevisiae* in response to different oxidants at 3 min, Figure S2: Growth curve analysis of *S. cerevisiae* under increasing oxidative stress.
