# Supplementary material for: Comparative Proteomics Analysis Reveals Unique Early Signaling Response of Saccharomyces cerevisiae to Oxidants with Different Mechanism of Action
Source: Int J Mol Sci. 2020 Dec 26;22(1):167. doi: 10.3390/ijms22010167 (PMC7795614; doi:10.3390/ijms22010167)
Supplement: Supplementary file 1 [file ijms-22-00167-s001.zip › ijms-1033713_FiguresS1-S2.docx]

**
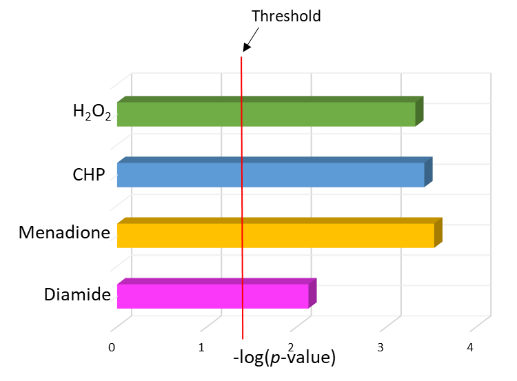
**

**Figure S1.** TOR signaling in *S. cerevisiae* in response to different oxidants at 3 min. Significance expressed in -log (*p*-value). The threshold: the minimum significance level scored as −log (*p*-value) from Fisher’s exact test, was set to 1.3. Pathway analysis was achieved using IPA^®^ based on global untargeted proteomics.


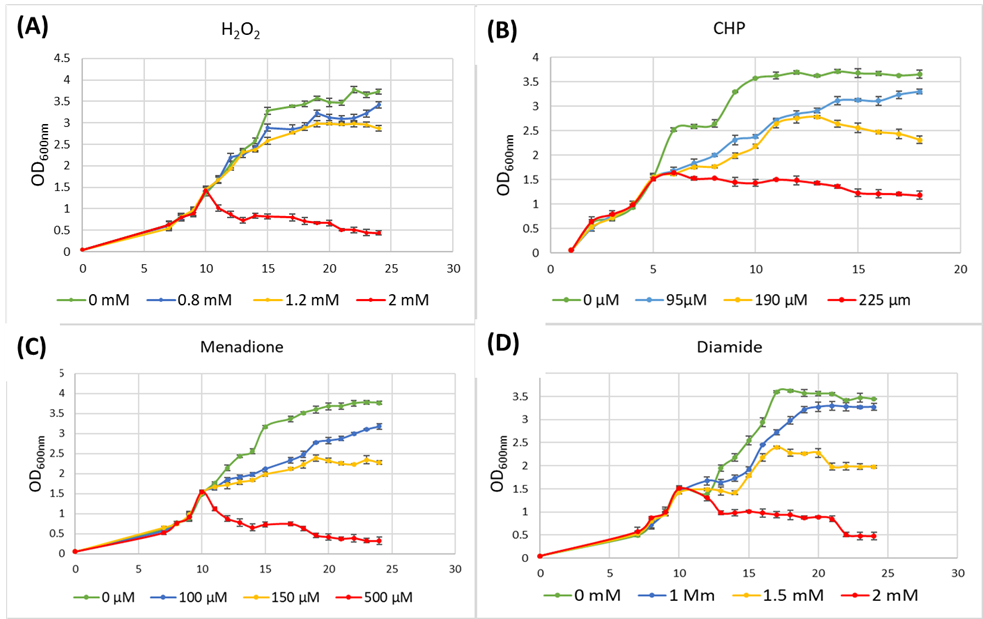


**Figure S2.** Growth curve analysis of S. cerevisiae under increasing oxidative stress. Cells were grown aerobically in minimal medium. At an OD at 600nm=1.5 (exponential growth phase) different concentrations of the oxidants were added to the cultures. (**A**) H2O2 (0 mM, 0.8 mM, 1.2 mM, and 2 mM), (**B**) CHP ( 0 µM, 95 µM, 190 µM and 225 µM), (**C**) menadione (0 µM, 100 µM, 150 µM, and 500 µM) and (**D**) diamide (0 mM, 1mM, 1.5 mM, and 2 mM). The curve represents the average of the replicates (n=3).
